# Childhood reading problems and cognitive ageing across mid to later life

**DOI:** 10.1136/jech-2020-215735

**Published:** 2021-07-06

**Authors:** Amber John, Josh Stott, Marcus Richards

**Affiliations:** 1 ADAPT Lab, Research Department of Clinical, Educational, and Health Psychology, UCL, London, UK; 2 MRC Unit for Lifelong Health and Ageing at UCL, UCL, London, UK

**Keywords:** longitudinal studies, cognition, ageing, cohort studies

## Abstract

**Background:**

Little research has investigated long-term associations of childhood reading with cognitive ageing. The aim of this study was to test longitudinal associations between childhood reading problems and cognitive function from mid-adulthood (age 43) to early old age (age 69), and whether associations were mediated by education.

**Methods:**

Data were from the MRC National Survey of Health and Development, a prospective population-based birth cohort. Reading problems were measured at age 11 using a reading test. Verbal memory and processing speed were measured at ages 43, 53, 60–64 and 69 and Addenbrooke’s Cognitive Examination (ACE) was administered at age 69. Linear mixed models and path analyses were used to test: (1) associations between reading problems and verbal memory and processing speed trajectories; (2) associations between reading problems and ACE-III scores; (3) whether associations were mediated by education.

**Results:**

Reading problems were associated with poorer verbal memory at intercept but not rate of decline (N=1726), and were not associated with processing speed intercept or decline (N=1730). There were higher rates of scores below ACE-III clinical thresholds (<82 and <88) in people with reading problems compared with those without. Reading problems were associated with poorer total ACE-III scores and all domain scores at age 69 (N=1699). Associations were partly mediated by education.

**Conclusion:**

Reading problems in childhood were associated with poorer cognitive function in early old age, and associations were partly mediated by education.

## Introduction

Reading problems are common, with estimates showing that 2%–15% of the population are affected.^1^ Research has shown that reading problems are stable over time and show strong persistence from childhood through to midlife.^2^ Evidence shows that reading problems in childhood can be associated with negative outcomes in adulthood, including lower income, poorer self-esteem and higher rates of psychiatric problems.^3–6^ Additionally, childhood reading problems have a clear impact on cognitive functioning across a range of different domains during childhood.^7^ However, little research has investigated the long-term association of childhood reading with cognitive ageing, and how any such association operates across a longer period over the life course. Previous research using data from the National Survey of Health and Development has shown that cognitive ability in childhood is significantly associated with faster decline from age 43 to 53.^8^ In addition, further research has shown that in the Lothian birth cohort 1921, childhood cognitive function did not significantly predict cognitive decline from age 79 to 97.^9^ A recent systematic review has also shown that childhood intelligence may not be associated with cognitive decline, and evidence was inconclusive from the association between childhood intelligence and dementia risk due to inconsistent findings.^10^ The current study extends findings from previous research by testing how childhood reading problems in the face of normal general cognitive ability are associated with later cognitive function and decline, whereas previous research has primarily focused on general cognitive ability in childhood. This study also extends previous findings by testing associations over a longer follow-up period of cognitive decline in adulthood (over a period of 26 years from midlife to early old age).

Moreover, it is currently unclear which processes potentially underlie associations between childhood reading and cognitive ageing outcomes. Research has shown that childhood reading problems are associated with lower education in adulthood.^3 4^ Additionally, education is also a strong predictor of adult cognitive function^11^ and risk of dementia.^12^ It is therefore plausible that any association between childhood reading problems and poorer cognitive ageing is at least partly mediated by education. However, to our knowledge no research has directly tested either element of this hypothesis. To do so we used data from a large and nationally representative British birth cohort of people of European heritage born in England, Scotland and Wales during 1946. Therefore, the primary aim of this research was to investigate longitudinal associations between childhood reading problems and cognitive function from mid-adulthood (age 43) to early old age (age 69). The secondary aim of this research was to test education as a potential process underlying these associations.

## Method

### Participants

Data were used from the MRC National Survey of Health and Development (NSHD, the British 1946 birth cohort). The sample originally comprised 5362 people born in England, Scotland and Wales during 1 week of 1946. Details about methods of data collection and participation rates at each sweep are published and available online.^13–15^ Missing data were handled using FIML. The analytic sample for path models was 1699, and for linear mixed models was 1726 for verbal memory and 1730 for processing speed.

### Measures

#### Reading problems

At age 11, all participating cohort members (N=4281, 79.8% of original sample) completed a general cognitive ability test, which consisted of 80 verbal and non-verbal reasoning items. At the same age, participants completed a reading test, in which cohort members pronounced 50 words of increasing difficulty, ranging from easy (eg, egg, book) to difficult (eg, extraneous, ophthalmic). Based on procedures defined and published on using this cohort, reading problems were defined as a reading score 1.5 SD below the mean with a general cognitive ability score at least 70% of the maximum.^16^ As an additional analysis, the score on the reading test was also used as a continuous measure (excluding cohort members with a general cognitive ability score of below 70, which is indicative of learning disability).

#### Cognitive function

Repeated measures of verbal memory and processing speed were available at age 43, 53, 60–64 and 69. Verbal memory was assessed using three trials of 15-item word recall tasks, in which cohort members recalled as many words as possible from a word list presented aurally. Verbal memory was derived by summing the scores across the three trials (max. 45). Processing speed was assessed using a letter cancellation task, in which cohort members used a timed 1 min period to cross out target letters randomly distributed within a grid of letters. The score reflects the total number of letters searched, with a possible range of 0–600 at ages 53, 60–64 and 69, and a possible range of 0–450 at age 43. Trajectories of cognitive function from mid to later life (age 43–69) in this cohort have been described.^17^ At age 69, cohort members completed the Addenbrooke’s Cognitive Examination III (ACE-III) as a measure of cognitive state. The lower threshold for screening potentially clinically significant cognitive impairment is 82 (of a possible 100) and the upper threshold is 88. The ACE-III measure comprises five subdomains: attention/orientation (18 points), verbal fluency (14 points), memory (26 points), language (26 points) and visuospatial function (16 points).^18^ Both the overall ACE-III score and the subdomains were used as outcomes in analyses.

#### Covariates

Covariates were selected based on known associations with cognitive function and/or reading problems. Covariates were sex, education, socioeconomic position in childhood and adulthood, and affective symptoms. A model was also run additionally adjusting for childhood general cognitive ability. Education was measured as the highest qualification achieved by the age of 26, coded as none (0), vocational or GCSE (1), or A-Level or higher (2). Socioeconomic position in childhood was derived from father’s social class when the cohort member was 4, 11 or 15. Socioeconomic position in adulthood was derived from social class of the cohort member at ages 26, 36, 43 and 53. Both socioeconomic position measures were coded into six categories according to the Registrar General (professional (1), intermediate (2), skilled non-manual (3), skilled manual (4), partly skilled (5), unskilled (6)). The General Health Questionnaire (GHQ-28) at age 69 was used as a measure of affective symptoms (possible range 0–28). Childhood cognition was assessed at ages 8, 11 and 15 using a general cognitive ability test, comprising 80 verbal and non-verbal reasoning items. Standardised scores at each age were used. Where information was missing at age 8, the score at age 11 was used. Where information was missing at both ages 8 and 11, the score at age 15 was used. The correlation matrix of all key predictors and covariates is presented in online supplemental table 1.

10.1136/jech-2020-215735.supp1 Supplementary data

### Statistical analysis

Longitudinal associations between reading problems at age 11 and cognitive outcomes were modelled using two complementary analyses. First, linear mixed models were fit to model memory and processing speed trajectories from middle to late adulthood. Intercepts (age 43) and slopes (from age 43 through 69) were random and an unstructured covariance structure was assumed, as previously applied in NSHD.^17^ Waves of cognitive data collection from age 43 to 69 was the time variable in analyses. In previous research, the quadratic model was the best fit for both memory and processing speed data compared with the linear model; therefore, the quadratic model was used for all subsequent analyses in this study. Reading problems at age 11 were used to predict cognitive trajectories. In order to test whether reading problems are associated with cognitive decline later in the life course (ie, starting from early old age, rather than midlife), a further subanalysis was conducted in which change scores were generated at the latter two time points (age 60–64 to 69) for both memory and processing speed. Linear regression was used to test associations between childhood reading problems and change scores in early old age.

Reading problems in childhood were then cross-tabbed against the clinically validated lower and upper thresholds (82 and 88) on the overall ACE-III score, and a χ^2^ test was used to test whether people with reading problems in childhood had significantly higher rates of scores below clinical thresholds on the ACE-III. Next, path models were conducted to test whether reading problems at age 11 were associated with cognitive function at age 69, as measured by ACE-III and its five subdomains (attention and orientation, verbal fluency, memory, language and visuospatial function). To test for the potential mediating role of education, a model was run including education as an indirect pathway between (1) reading problems and cognitive function at age 69 (total score and the five subdomains) and (2) reading problems and cognitive function at age 43. Model fit was assessed using standard fit statistics: χ^2^ goodness of fit test (p>0.05), Comparative Fit Index (CFI) (>0.90), Tucker Lewis Index (TLI) (>0.90) and root mean square error of approximation (RMSEA) (0.08).

Analyses were adjusted for the covariates. Models were also rerun adjusting for general cognitive ability in childhood. Models were also rerun using the continuous reading score as a predictor. No significant interaction between reading problems and sex were observed. Therefore, sex was included in models as a covariate rather than a stratifying variable. Missing data were addressed using full information maximum likelihood (FIML).^19^ Analyses were run in Mplus, Stata v16 and RStudio.

## Results

### Missing data and demographic information

There were 1281 people who completed all cognitive measures (memory and processing speed at ages 43, 53, 60–64 and 69 and ACE-III at age 69). Of this sample, 1102 people (86.03%) had a reading score measure available at age 11. Finally, within this group, 1051 people (95.37%) also had complete information for all key covariates (sex, childhood socioeconomic position, adulthood socioeconomic position, education, adult affective symptoms). Those with missing data (N=4311) had significantly lower ACE-III scores (t(1381.53)=−3.37, p=0.001), poorer memory function at all ages (age 43: t(2320.05)=−9.77, p<0.001; age 53: t(2338.73)=−8.87, p<0.001; age 60–64: t(2148)=−4.40, p<0.001; age 69: t(2072)=−4.18, p<0.001), were more likely to have reading problems (χ^2^(1)=30.12, p<0.001), lower education (χ^2^(2)=107.07, p<0.001), be of lower childhood (χ^2^(5)=42.87, p<0.001) and adulthood socioeconomic position (χ^2^(5)=93.91, p<0.001), be male (χ^2^(1)=9.94, p=0.002), and have poorer childhood cognition (t(1968.95)=−9.33, p<0.001). However, people with missing data did not differ from those with complete data on adulthood affective symptoms (t(2123)=1.35, p=0.18), or processing speed scores at any time point (age 43: t(3129)=−0.82, p=0.41; age 53: t(2930)=−0.70, p=0.48; age 60–64: t(2180)=−0.48, p=0.63; age 69: t(2109)=−0.57, p=0.57) (online supplemental table 2). Missing data were handled using FIML. Demographic information was explored for the 1051 people with complete information for all key measures and covariates (table 1).

**Table 1:** Demographic information of the sample with complete information for all key variables and covariates

	Mean (SD) Total sample	Mean (SD) Sample without reading problems	Mean (SD) Sample with reading problems	N (%) Total sample	N (%) Sample without reading problems	N (%) Sample with reading problems
Verbal memory						
Age 43	26.21 (5.90)	26.55 (5.69)	18.76 (5.55)	–	–	–
Age 53	25.25 (5.89)	25.55 (5.75)	18.74 (5.27)	–	–	–
Age 60–64	24.85 (6.00)	25.15 (5.84)	18.22 (5.65)	–	–	–
Age 69	22.71 (6.04)	23.01 (5.89)	16.22 (5.57)	–	–	–
Processing speed						
Age 43	343.44 (74.23)	343.29 (73.78)	346.61 (84.29)	–	–	–
Age 53	282.39 (73.05)	282.93 (73.69)	270.67 (56.71)	–	–	–
Age 60–64	267.48 (70.20)	267.36 (70.30)	270.17 (68.65)	–	–	–
Age 69	263.22 (71.67)	262.94 (70.79)	269.43 (89.49)	–	–	–
ACE-III total score	91.94 (5.59)	92.28 (5.30)	84.43 (6.41)	–	–	–
ACE-III fluency	11.09 (2.06)	11.15 (2.05)	9.65 (1.95)	–	–	–
ACE-III language	25.33 (1.06)	25.40 (0.97)	23.93 (1.78)	–	–	–
ACE-III attention	16.78 (1.79)	16.83 (1.74)	15.76 (2.46)	–	–	–
ACE-III memory	23.62 (2.67)	23.76 (2.57)	20.65 (3.02)	–	–	–
ACE-III visuospatial	15.12 (1.22)	15.15 (1.20)	14.43 (1.41)	–	–	–
Reading problems						
Yes	–	–	–	46 (4.4)	–	–
No	–	–	–	1005 (95.6)	–	–
Reading problems (continuous)	38.92 (8.63)	40.04 (6.93)	14.46 (5.12)	–	–	–
Sex						
Male	–	–	–	506 (48.1)	482 (48.0)	24 (52.2)
Female	–	–	–	545 (51.9)	5.23 (52.0)	22 (47.8)
Childhood cognition	0.19 (0.78)	0.25 (0.75)	−0.95 (0.49)	–	–	–
Childhood socioeconomic position						
Professional	–	–	–	85 (8.1)	84 (8.4)	1 (2.2)
Intermediate	–	–	–	238 (22.6)	234 (23.3)	4 (8.7)
Skilled non-manual	–	–	–	189 (18.0)	186 (18.5)	3 (6.5)
Skilled manual	–	–	–	334 (31.8)	312 (31.0)	22 (47.8)
Partly skilled	–	–	–	162 (15.4)	153 (15.2)	9 (19.6)
Unskilled	–	–	–	43 (4.1)	36 (3.6)	7 (15.2)
Adulthood socioeconomic position						
Professional	–	–	–	94 (8.9)	93 (9.3)	1 (2.2)
Intermediate	–	–	–	449 (42.7)	440 (43.8)	9 (19.6)
Skilled non-manual	–	–	–	241 (22.9)	233 (23.2)	8 (17.4)
Skilled manual	–	–	–	145 (13.8)	127 (12.6)	18 (39.1)
Partly skilled	–	–	–	96 (9.1)	88 (8.8)	8 (17.4)
Unskilled	–	–	–	26 (2.5)	24 (2.4)	2 (4.3)
Education						
None	–	–	–	281 (26.7)	252 (25.1)	29 (63.0)
Vocational or GCSE	–	–	–	319 (30.4)	307 (30.5)	12 (26.1)
A-level or higher	–	–	–	451 (42.9)	446 (44.4)	5 (10.9)
Adulthood affective symptoms	1.64 (3.38)	1.61 (3.28)	2.30 (5.06)	–	–	–

ACE-III, Addenbrooke’s Cognitive Examination III; GCSE, General Certificate of Secondary Education.

### Reading problems and cognitive trajectories from age 43 to 69

**Figure 1:**
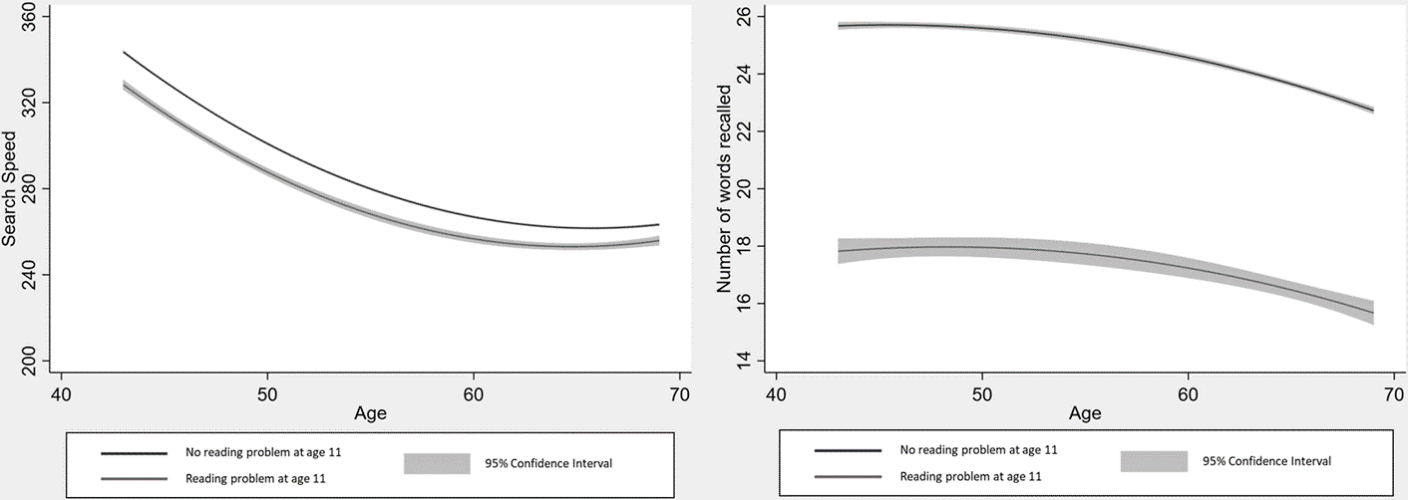
Cognitive (memory and processing speed) trajectories for people with and without reading problems at age 11.

Fully adjusted linear mixed models revealed that reading problems at age 11 were significantly associated with poorer verbal memory (b=−5.65, SE=1.30, p<0.001) at intercept, but not with the rate of decline from age 43 to 69 (b=0.03, SE=0.02, p=0.22) (Akaike's Information Criteria AIC: 37 301.39; Bayesian Information Criteria BIC: 37 405.01). Additionally, reading problems were not significantly associated with trajectories of processing speed (intercept: b=−8.05, SE=17.81, p=0.65; slope: b=0.22, SE=0.30, p=0.46) (AIC: 71 520.78; BIC: 71 624.44) (table 2, figure 1). Models including childhood cognition as a covariate, showed the same pattern of associations (online supplemental table 3).

**Table 2:** Linear mixed models to test effects of reading problems at age 11 on memory and processing speed trajectories from age 43 to 69

	Verbal memory (N=1726)	Processing speed (N=1730)
Intercept		
Reading problems	−5.65 (1.30), <0.001*	−8.05 (17.81), 0.65
Sex	1.42 (0.58), 0.02	40.98 (8.27), <0.001
Education	2.75 (0.41), <0.001	18.44 (5.90), 0.002
Childhood SEP†	0.17 (0.24), 0.49	0.81 (3.38), 0.81
Adulthood SEP†	−1.11 (0.27), <0.001	−0.15 (3.81), 0.97
Adulthood affective symptoms	0.11 (0.08), 0.20	1.10 (1.20), 0.36
Slope		
Reading problems	0.03 (0.02), 0.22	0.22 (0.30), 0.46
Sex	0.02 (0.01), 0.04	−0.42 (0.14), 0.002
Education	−0.01 (0.01), 0.07	−0.15 (0.10), 0.11
Childhood SEP†	−0.01 (0.004), 0.02	−0.06 (0.06), 0.28
Adulthood SEP†	0.005 (0.004), 0.25	−0.04 (0.06), 0.55
Adulthood affective symptoms	−0.004 (0.001), 0.007	−0.03 (0.02), 0.14

*b (SE), p.

†Reverse coded.

SEP, Socioeconomic position.

Models were rerun using a continuous measure of reading as a predictor of cognitive trajectories. Results remained consistent; higher continuous reading scores predicted higher verbal memory at baseline (b=0.26, SE=0.04, p<0.001), but not rate of decline from age 43 to 69 (b=−0.001, SE=0.001, p=0.08). Reading score was not associated with processing speed trajectories (intercept: b=−0.13, SE=0.52, p=0.80; slope: b=0.002, SE=0.01, p=0.81).

Linear regression models were run using reading problems to predict cognitive function change scores from age 60–64 to 69. Fully adjusted models revealed that childhood reading problems did not significantly predict change in verbal memory (b=−0.03, SE=0.63, p=0.96) or processing speed (b=11.79, SE=7.93, p=0.14) from age 60–64 to 69.

### Reading problems and the ACE at age 69

There were higher rates of scores <82 on ACE-III in people who had reading problems (<82 score: 32.95%; >82 score: 67.05%) than people without reading problems (<82 score: 4.75%; >82 score: 95.25%) (χ^2^(1)=105.29, p<0.001). For the upper ACE-III threshold (88), ratios were <88 score: 61.36%; >88 score: 38.64% in people with reading problems compared with <88 score: 18.43%; >88 score: 81.57%) in people without reading problems (χ^2^(1)=89.65, p<0.001).

The fully adjusted linear regression model results showed that reading problems were significantly associated with lower ACE-III total score at age 69 (b=−5.14, SE=0.60, p<0.001). The fully adjusted path model including ACE-III subdomain scores was a good fit to the data (χ^2^(1)=2.63, p=0.11; CFI=0.999; TLI=0.953; RMSEA=0.031). Results revealed that reading problems were significantly associated with lower scores on all ACE-III domains (fluency: b=−1.19, SE=0.21, p<0.001; language: b=−1.39, SE=0.12, p*<*0.001; attention: b=−0.45, SE=0.20, p=0.03; memory: b=−1.95, SE=0.30, p<0.001; visuospatial: b=−0.43, SE=0.14, p=0.002) (table 3; online supplemental figure 1).

**Table 3:** Path model to test effects of reading problems at age 11 on Addenbrooke’s Cognitive Examination III scores at age 69

	Fluency	Language	Attention	Memory	Visuospatial
Reading problems	−1.19 (0.21), <0.001*	−1.39 (0.12), <0.001	−0.45 (0.20), 0.03	−1.95 (0.30), <0.001	−0.43 (0.14), 0.002
	−0.13 (0.02), <0.001†	−0.29 (0.02), <0.001	0.06 (0.03), 0.03	−0.16 (0.03), <0.001	0.08 (0.03), 0.002
Sex	0.35 (0.10), <0.001	0.08 (0.06), 0.16	−0.31 (0.09), <0.001	0.68 (0.14), <0.001	−0.11 (0.06), 0.08
	0.08 (0.02), <0.001	0.03 (0.02), 0.16	−0.09 (0.03), 0.001	0.12 (0.02), <0.001	−0.05 (0.03), 0.08
Education	0.49 (0.07), <0.001	0.22 (0.04), <0.001	0.20 (0.07), 0.003	0.64 (0.10), <0.001	0.30 (0.05), <0.001
	0.19 (0.03), <0.001	0.16 (0.03), <0.001	0.09 (0.03), 0.003	0.19 (0.03), <0.001	0.20 (0.03), <0.001
Childhood SEP	−0.14 (0.04), 0.001	−0.06 (0.02), 0.01	−0.02 (0.04), 0.55	−0.16 (0.06), 0.006	−0.07 (0.03), 0.009
	−0.08 (0.03), 0.001	−0.06 (0.03), 0.01	−0.02 (0.03), 0.55	−0.07 (0.03), 0.006	−0.07 (0.03), 0.008
Adulthood SEP	−0.20 (0.05), <0.001	−0.11 (0.03), <0.001	−0.11 (0.04), 0.02	−0.27 (0.06), <0.001	−0.11 (0.03), <0.001
	−0.11 (0.03), <0.001	−0.12 (0.03), <0.001	−0.07 (0.03), 0.02	0.12 (0.03), <0.001	−0.11 (0.03), <0.001
Affective symptoms	−0.02 (0.01), 0.29	−0.02 (0.01), 0.02	−0.01 (0.01), 0.57	−0.08 (0.02), <0.001	−0.01 (0.01), 0.12
	−0.02 (0.02), 0.29	−0.05 (0.02), 0.02	−0.02 (0.03), 0.57	−0.09 (0.02), <0.001	−0.04 (0.03), 0.12

Model fit statistics: N=1699; χ^2^(1)=2.63, p=0.11; Comparative Fit Index=0.999; Tucker Lewis Index=0.953; root mean square error of approximation=0.031.

Missing data handled using full information maximum likelihood.

*b (SE), p.

†Standardised beta presented on second rows.

SEP, Socioeconomic position.

Next, path models were conducted including an indirect pathway from reading problems to cognitive outcomes through education. The model using the ACE-III total score showed a significant direct effect of reading problems on ACE-III total (b=−5.18, SE=0.60, p<0.001). There was also a significant indirect effect of reading problems on total ACE-III score through education (b=−0.74, SE=0.15, p<0.001). The model including the domain scores fit the data well (χ^2^(1)=2.39, p=0.12; CFI=0.999; TLI=0.970; RMSEA=0.028). Results revealed significant direct effects of reading problems on fluency (b=−1.20, SE=0.21, p=0.001), language (b=−1.39, SE=0.12, p<0.001), attention (b=−0.45, SE=0.20, p=0.03), memory (b=−1.98, SE=0.30, p<0.001) and visuospatial ability (b=−0.44, SE=0.14, p=0.001). There were also significant indirect effects of reading problems on all cognitive domains through the education pathway (fluency: b=−0.20, SE=0.05, p<0.001; language: b=−0.09, SE=0.02, p<0.001; attention: b=−0.08, SE=0.03, p=0.01; memory: b=−0.26, SE=0.06, p<0.001; visuospatial: b=−0.13, SE=0.03, p<0.001) (online supplemental table 4). The path model testing effects of reading problems on memory and search speed at age 43 through education showed significant direct effects of reading problems on memory (b=−4.53, SE=0.60, p<0.001), but not processing speed (b=6.23, SE=7.79, p=0.42). There were significant indirect effects through education for both memory (b=−0.87, SE=0.17, p<0.001) and processing speed (b=−5.04, SE=1.38, p<0.001).

Models including childhood cognition as an additional covariate showed that reading problems in childhood significantly predicted total ACE-III scores (b=−3.59, SE=0.60, p<0.001). The path model was a good fit to the data (χ^2^(1)=1.18, p=0.28; CFI=1.000; TLI=0.995; RMSEA=0.010). Reading problems were significantly associated with poorer fluency (b=−0.71, SE=0.21, p=0.001), language (b=−1.19, SE=0.12, p<0.001), and memory (b=−1.48, SE=0.31, p<0.001) scores at age 69, but not attention (b=−0.27, SE=0.21, p=0.19) or visuospatial (b=−0.21, SE=0.14, p=0.13) scores (online supplemental table 5). Models including education as an indirect pathway (χ^2^(1)=1.00, p=0.32; CFI=1.000; TLI=1.000; RMSEA=0.001) revealed that there were significant effects of education on all cognitive domains (fluency: b=0.30, SE=0.07, p<0.001; language: b=0.14, SE=0.04, p=0.001; memory: b=0.45, SE=0.10, p<0.001; visuospatial: b=0.22, SE=0.05, p<0.001), except for attention (b=0.12, SE=0.07, p=0.08). However, childhood reading problems were not significantly associated with education (b=−0.14, SE=0.07, p=0.05). Therefore, there were no significant overall indirect effects of reading problems on any cognitive domain through the education pathway (fluency: b=−0.04, SE=0.02, p=0.08; language: b=−0.02, SE=0.01, p=0.09; attention: b=−0.02, SE=0.01, p=0.20; memory: b=−0.06, SE=0.03, p=0.08; visuospatial: b=−0.03, SE=0.02, p=0.08) (online supplemental table 6).

Path models were rerun using the continuous measure of reading as a predictor of cognitive outcomes. Linear regression showed that reading score was significantly associated with total ACE-III score at age 69 (b=0.23, SE=0.02, p<0.001). The fully adjusted path model including the continuous reading score as a predictor of ACE-III domain scores fit the data well (χ^2^(1)=0.84, p=0.36; CFI=1.000; TLI=1.005; RMSEA=0.000). Results revealed that higher reading scores were significantly associated with better cognitive function in all domains at age 69 (fluency: b=0.06, SE=0.01, p<0.001; language: b=0.05, SE=0.01, p<0.001; attention: b=0.02, SE=0.01, p<0.001; memory: b=0.09, SE=0.01, p<0.001; visuospatial: b=0.03, SE=0.004, p<0.001). The model testing indirect effects of continuous reading scores on cognitive outcomes at age 69 showed that there were significant direct effects of reading problems on all cognitive domains (fluency: b=0.06, SE=0.01, p<0.001; language: b=0.05, SE=0.003, p<0.001; attention: b=0.02, SE=0.01, p<0.001; memory: b=0.09, SE=0.01, p<0.001; visuospatial: b=0.03, SE=0.004, p<0.001). There were significant indirect effects through education for all domains (fluency: b=0.01, SE=0.002, p<0.001; language: b=0.003, SE=0.001, p=0.005; memory: b=0.01, SE=0.003, p<0.001; visuospatial: b=0.01, SE=0.001, p<0.001), except for attention (b=0.003, SE=0.002, p=0.07) (online supplemental table 7).

## Discussion

### Summary of findings

These results show that reading problems at age 11 were associated with poorer memory at age 43, but not the rate of decline from 43 to 69. Reading problems were not associated with intercept or slope of processing speed. Additionally, there were significantly higher rates of scores below the clinical thresholds of ACE-III in people with reading problems. Reading problems were associated with lower scores on all domains and total score on the ACE-III at age 69. These associations were partially mediated by education. After adjustment for cognitive ability in childhood, reading problems were significantly associated with poorer scores in verbal cognitive functions in early old age (fluency, language and memory), but not non-verbal (attention and visuospatial).

Developmental reading problems are primarily characterised by an underlying deficit in phonological processing, which interferes with grapheme-phoneme mapping.^20^ This impairment in phonological processing may potentially explain associations observed in this study between reading problems in childhood and poorer cognitive test scores in early old age. For example, verbal fluency comprises the ability to retrieve words based on semantic or phonemic criteria within a specified time limit. There has been consistent evidence to suggest that people with reading problems in childhood retrieve fewer words in verbal fluency tasks than those without reading problems, and that this effect is more prominent for phonemic fluency tasks than for semantic.^21^ This may be driven by the underlying phonological processing characteristic of developmental reading problems. In addition, reading problems in childhood have also been associated with poorer verbal memory.^22^ Previous research has suggested this may be due to difficulties in phonetic encoding, which can also stem from the deficits in phonological processing associated with developmental reading problems.^23^ In addition, the language component of the ACE-III contains items which can be directly affected by reading problems. For example, this includes items testing reading and writing, which both can be affected by the relevant phonological processing impairment^24^ as well as fluency and verbal memory, which can be indirectly affected as described earlier.

Results from this study complement previous research using other cohorts, which has shown that reading problems are associated with poorer functioning across several cognitive domains in childhood, including the domains observed in this study. These results also extend this previous research by demonstrating that cognitive difficulties associated with childhood reading problems can persist across the life course into early old age. These results show that cognitive dysfunction associated with childhood reading problems can be observed towards the seventh decade. Findings extend previous results from studies using NSHD and Lothian birth cohort 1921 data, which tested associations between general cognitive ability in childhood and cognitive decline in adulthood by showing that reading problems in childhood independent of general cognitive ability are associated with verbal cognitive function in early old age.

Additionally, these results extend previous research using other cohorts by showing that associations between reading problems and cognitive deficits in early old age are partially mediated by education. Specifically, childhood reading problems are associated with lower education, which is in turn associated with poorer cognitive function at age 69. However, even when including the indirect pathway through education in the model, direct effects between childhood reading problems and cognitive function at age 69 remained statistically significant. There may be other important mechanisms in this association. For example, childhood reading problems have been linked with both income^3^ and self-esteem,^5^ both of which may also be associated with cognitive ageing outcomes. There may also be a neurodevelopmental path, supported by research in this cohort showing delayed developmental milestones in children with reading problems.^16^ It is important to note that the mediating effect of education was no longer significant after additionally adjusting for childhood cognition, although we have shown elsewhere that education is associated with later cognitive function even when childhood cognition is controlled.^11 25^

Childhood reading problems are associated with lower scores on the ACE-III, a commonly used screening tool for cognitive impairment. Around 33% of the sample with reading problems fell below the lower ACE-III threshold for dementia as opposed to 5% in the sample without such problems. There are several potential explanations which may underlie this finding. First, reading problems in childhood lead to a greater risk of poorer cognitive outcomes in part due to lower education, which may explain the apparent cross-modal associations with the ACE-III subscores, as opposed to a specific verbal effect. The lack of association with cognitive decline would count against reading problems being a specific risk factor for dementia, although this needs to be viewed in the context of the relatively young age of this sample. These findings are important because increasing current understanding of early risk factors for cognitive ageing can be of potential benefit for building better predictive models.

### Strengths and limitations

Strengths of this study include the use of a large population-based cohort of people, with reading problems derived from tests administered in childhood. This study benefitted from a long follow-up period of 58 years, allowing long-term associations between reading problems and cognitive ageing to be modelled.

However, there are missing data in this sample and cohort members with reading problems and poorer cognitive function were more likely to drop out. In this study, missing data were addressed using FIML. Estimates produced using FIML are less biased and more reliable than those produced complete case analysis, even in cases where missing data are non-ignorable.^11^ In addition, a distinction should be made between missing data and selective attrition in these data. In this study, memory and processing speed were measured repeatedly across four time points from age 43 to 69. A limitation, however, is that data are only available up to age 69, so cognitive trajectories later in the life course cannot be modelled. It is possible that effects of reading problems on cognitive decline may only be observed later in the life course. Continuing follow-up of cognitive measures in these data will allow these associations to be modelled in the future. It should also be noted that the definition of reading problems is limited by the data available in this cohort. Lower reading scores may reflect a range of different underlying conditions, including a developmental disorder, dyslexia or autism. It is possible that each of these underlying conditions may have differential effects on long-term cognitive outcomes. However, due to the data available, this could not be systematically tested in the current study.

## Conclusions

Reading problems in childhood were associated with poorer memory scores at age 43, but not rate of memory decline from 43 to 69. Reading problems were not associated with intercept or slope of processing speed. Additionally, reading problems were associated with lower ACE-III scores at age 69, and these associations were mediated by education. There were also significantly higher rates of people with reading problems in childhood who scored below the clinical threshold on the ACE-III than people without reading problems. In conclusion, childhood reading problems are associated with poorer cognitive skills but not decline in early older age, and this association is partly mediated by education.

**What is already known on this subject?**

- Reading problems in childhood are common and are associated with adverse socioeconomic and health outcomes. Reading problems are associated with poorer cognitive function in childhood and adolescence. However, the long-term association of childhood reading with cognitive ageing over the life course is currently unknown.

**What this study adds?**

- This study extends previous research by showing that reading problems in childhood are associated with cognitive function in early old age, and these associations are mediated by educational attainment. Additionally, people with childhood reading problems were more likely to score below the clinical threshold on a cognitive measure, routinely used in clinical settings to aid in diagnosis of dementia (Addenbrooke’s Cognitive Examination III). These findings are important because they highlight the long-lasting effects of childhood reading problems on cognitive health across the life-course over a follow-up period of six decades.

## Data Availability

Data are available upon reasonable request. Data are available upon application to MRC Unit for Lifelong Health and Ageing at UCL.
