# Supplementary material for: Childhood reading problems and cognitive ageing across mid to later life
Source: J Epidemiol Community Health. 2021 Jul 6;76(1):67–74. doi: 10.1136/jech-2020-215735 (PMC8666812; doi:10.1136/jech-2020-215735)
Supplement: Supplementary data [file jech-2020-215735supp001.pdf]

SUPPLEMENTARY MATERIALS

Supplementary Table 1: Correlation matrix of key predictors and covariates.

Supplementary Table 2: Missing data analysis.

Supplementary Table 3: Linear mixed models to test effects of reading problems at age 11 on memory and processing speed trajectories from age 43 to 69 (with childhood cognition as a covariate).

Supplementary Figure 1: Path model of reading problems at age 11 on ACE outcomes at age 69.

Supplementary Table 4: Path model showing direct effects of reading problems on ACE-III outcomes at age 69 and indirect effects through educational attainment.

Supplementary Table 5: Path model to test effects of reading problems at age 11 on ACE-III scores at age 69 (with childhood cognition as a covariate).

Supplementary Table 6: Path model showing direct effects of reading problems on ACE-III outcomes at age 69 and indirect effects through educational attainment (with childhood cognition as a covariate).

Supplementary Table 7: Path model showing direct effects of continuous reading problems on ACE-III outcomes at age 69 and indirect effects through educational attainment.

Supplementary Table 1: Correlation matrix of key predictors and covariates.

	**Reading Problems**	**Sex**	**Education**	**Childhood socioeconomic position**	**Adult socioeconomic position**	**Adult affective symptoms**	**Childhood cognition**
**Reading Problems**	1	-0.04, *p*=.007	-0.26, *p*<.001	0.18, *p*<.001	0.23, *p*<.001	0.05, *p*<.001	-0.41, *p*<.001
**Sex**	-0.04, *p*=.007	1	-0.07, *p*<.001	0.01, *p*<.64	0.09, *p*<.001	-0.06, *p*<.001	0.03, *p*=.08
**Education**	-0.26, *p*<.001	-0.07, *p*<.001	1	-0.43, *p*<.001	-0.53, *p*<.001	-0.18, *p*<.001	0.55, *p*<.001
**Childhood socioeconomic position**	0.18, *p*<.001	0.01, *p*<.64	-0.43, *p*<.001	1	0.34, *p*<.001	0.11, *p*<.001	-0.39, *p*<.001
**Adult socioeconomic position**	0.23, *p*<.001	0.09, *p*<.001	-0.53, *p*<.001	0.34, *p*<.001	1	0.18, *p*<.001	-0.46, *p*<.001
**Adult affective symptoms**	0.05, *p*<.001	-0.06, *p*<.001	-0.18, *p*<.001	0.11, *p*<.001	0.18, *p*<.001	1	-0.14, *p*<.001
**Childhood cognition**	-0.41, *p*<.001	0.03, *p*=.08	0.55, *p*<.001	-0.39, *p*<.001	-0.46, *p*<.001	-0.14, *p*<.001	1

Supplementary Table 2: Missing data analysis.

	t/X^2	df	p
Verbal memory			
Age 43	-9.77	2320.05	<.001
Age 53	-8.87	2338.73	<.001
Age 60-64	-4.40	2148	<.001
Age 69	-4.18	2072	<.001
Letter search speed			
Age 43	-0.82	3129	.41
Age 53	-0.70	2930	.48
Age 60-64	-0.48	2180	.63
Age 69	-0.57	2109	.57
ACE-III Total score	-3.37	1381.53	.001
Reading problems	30.12	1	<.001
Sex	9.94	1	.002
Childhood cognition	-9.33	1968.95	<.001
Childhood socioeconomic position	42.87	5	<.001
Adulthood socioeconomic position	93.91	5	<.001
Education	107.07	2	<.001
Adult affective symptoms	1.35	2123	.18

Supplementary Table 3: Linear mixed models to test effects of reading problems at age 11 on memory and processing speed trajectories from age 43 to 69 (with childhood cognition as a covariate).

	**Verbal memory (N=1726)**	**Processing speed (N=1730)**
Intercept		
Reading problems	-4.28 (1.34), .001*	-9.28 (18.44), .62
Sex	1.22 (0.58), .04	41.20 (8.30), <.001
Education	2.22 (0.43), <.001	18.93 (6.18), .002
Childhood cognition	1.80 (0.44), <.001	-1.68 (6.33), .79
Childhood SEP**	0.31 (0.24), .20	0.65 (3.42), .85
Adulthood SEP**	-0.92 (0.27), .001	-0.34 (3.88), .93
Adulthood affective symptoms	0.12 (0.08), .17	1.09 (1.20), .36
Slope		
Reading problems	0.03 (0.02), .21	0.33 (0.31), .28
Sex	0.02 (0.01), .05	-0.43 (0.14), .002
Education	-0.01 (0.01), .07	-0.20 (0.10), .05
Childhood cognition	0.001 (0.01), .88	0.15 (0.10), .16
Childhood SEP**	-0.01 (0.004), .03	-0.05 (0.06), .39
Adulthood SEP**	0.01 (0.005), .25	-0.02 (0.06), .74
Adulthood affective symptoms	-0.004 (0.001), .007	-0.03 (0.02), .14

* b (SE), *p*

** Reverse coded

Supplementary Figure 1: Path model of reading problems at age 11 on ACE outcomes at age 69

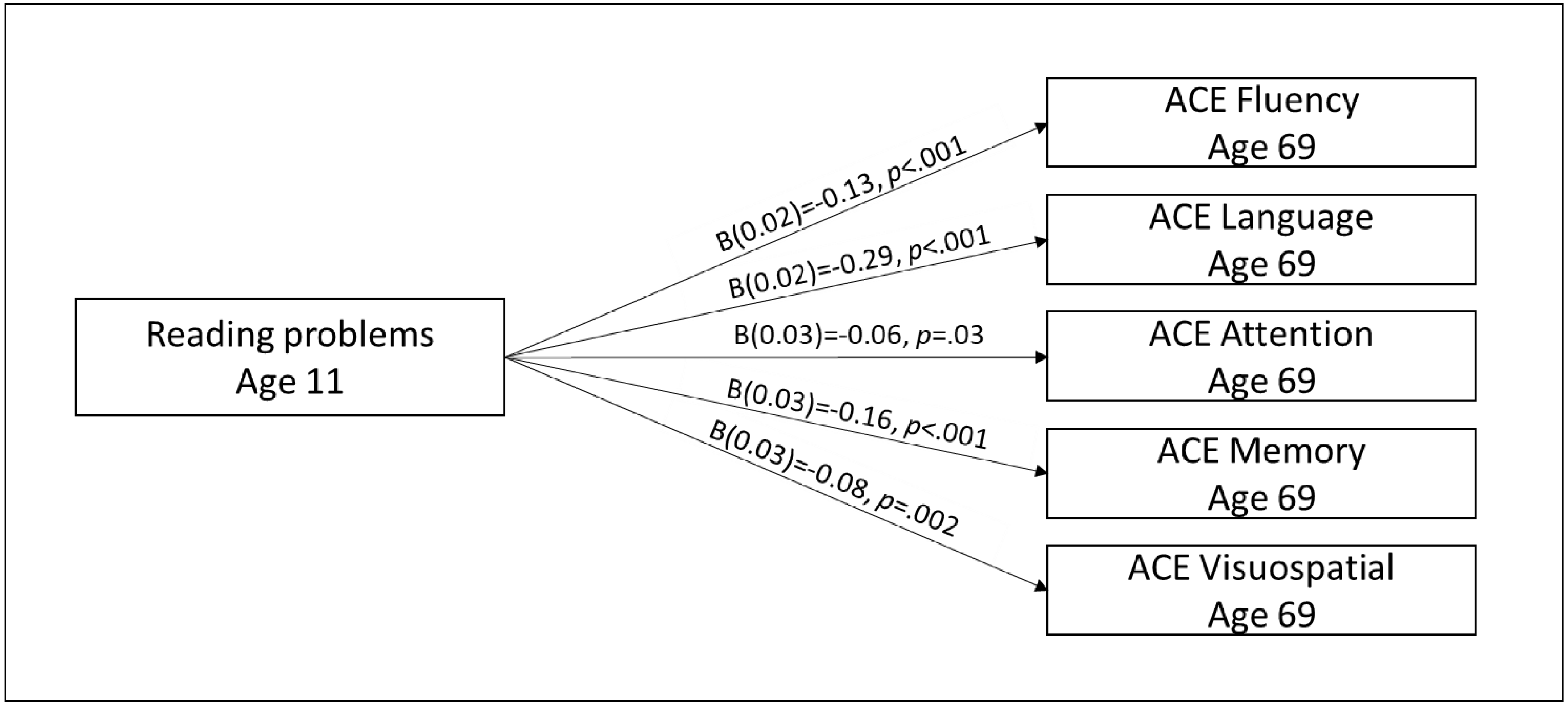

*Standardised beta presented.

*All pathways in the model were adjusted for all key covariates.

Supplementary Table 4: Path model showing direct effects of reading problems on ACE-III outcomes at age 69 and indirect effects through educational attainment.

	**Fluency**	**Language**	**Attention**	**Memory**	**Visuospatial**
Reading prob -> Cognition	-1.20 (0.21), <.001*	-1.39 (0.12), <.001	-0.45 (0.20), .03	-1.98 (0.30), <.001	-0.44 (0.14), .001
Reading prob -> Education -> Cognition	-0.20 (0.05), <.001	-0.09 (0.02), <.001	-0.08 (0.03), .01	-0.26 (0.06), <.001	-0.13 (0.03), <.001

Model fit statistics: N=1776; X^2 (1)=2.39, *p*=.12; CFI=0.999; TLI=0.970; RMSEA=0.028.

Missing data dealt with using FIML

* b (SE), *p*

Supplementary Table 5: Path model to test effects of reading problems at age 11 on ACE-III scores at age 69 (with childhood cognition as a covariate).

	**Fluency**	**Language**	**Attention**	**Memory**	**Visuospatial**
Reading problems	-0.71 (0.21), .001*	-1.19 (0.12), <.001	-0.27 (0.21), .19	-1.48 (0.31), <.001	-0.21 (0.14), .13
Sex	0.29 (0.10), .003	0.05 (0.05), .39	-0.34 (0.09), <.001	0.61 (0.14), <.001	-0.15 (0.06), .02
Education	0.30 (0.07), <.001	0.14 (0.04), .001	0.13 (0.07), .07	0.45 (0.10), <.001	0.22 (0.05), <.001
Childhood cognition	0.63 (0.07), <.001	0.27 (0.04), <.001	0.23 (0.07), .001	0.63 (0.11), <.001	0.29 (0.05), <.001
Childhood SEP	-0.08 (0.04), .03	-0.03 (0.02), .13	-0.01 (0.04), .91	-0.11 (0.06), .06	-0.05 (0.03), .09
Adulthood SEP	-0.12 (0.05), .006	-0.08 (0.03), .001	-0.08 (0.04), .08	-0.20 (0.07), .002	-0.08 (0.03), .007
Affective symptoms	-0.01 (0.01), .36	-0.02 (0.01), .03	-0.01 (0.01), .63	-0.07 (0.02), <.001	-0.01 (0.01), .16

* Model fit statistics: N=1699; X^2 (1)=1.18, *p*=.28; CFI=1.000; TLI=0.995; RMSEA=0.010.

* b (SE), *p*

Supplementary Table 6: Path model showing direct effects of reading problems on ACE-III outcomes at age 69 and indirect effects through educational attainment (with childhood cognition as a covariate).

	**Fluency**	**Language**	**Attention**	**Memory**	**Visuospatial**
Reading prob -> Cognition	-0.71 (0.21), .001*	-1.20 (0.12), <.001	-0.26 (0.21), .21	-1.50 (0.30), <.001	-0.22 (0.14), .11
Reading prob -> Education -> Cognition	-0.04 (0.02), .08	-0.02 (0.01), .09	-0.02 (0.01), .20	-0.06 (0.03), .08	-0.03 (0.02), .08

* Model fit statistics: N=1776; X^2 (1)=1.00, *p*=.32; CFI=1.000; TLI=1.000; RMSEA=0.001.

* b (SE), *p*

Supplementary Table 7: Path model showing direct effects of continuous reading problems on ACE-III outcomes at age 69 and indirect effects through educational attainment.

	**Fluency**	**Language**	**Attention**	**Memory**	**Visuospatial**
Reading prob -> Cognition	0.06 (0.01), <.001*	0.05 (0.003), <.001	0.02 (0.01), <.001	0.09 (0.01), <.001	0.03 (0.004), <.001
Reading prob -> Education -> Cognition	0.01 (0.002), <.001	0.003 (0.001), .005	0.003 (0.002), .07	0.01 (0.003), <.001	0.01 (0.001), <.001

Model fit statistics: N=1776; X^2 (1)=0.78, *p*=.38; CFI=1.000; TLI=1.004; RMSEA=0.000.

Missing data dealt with using FIML

* b (SE), *p*
